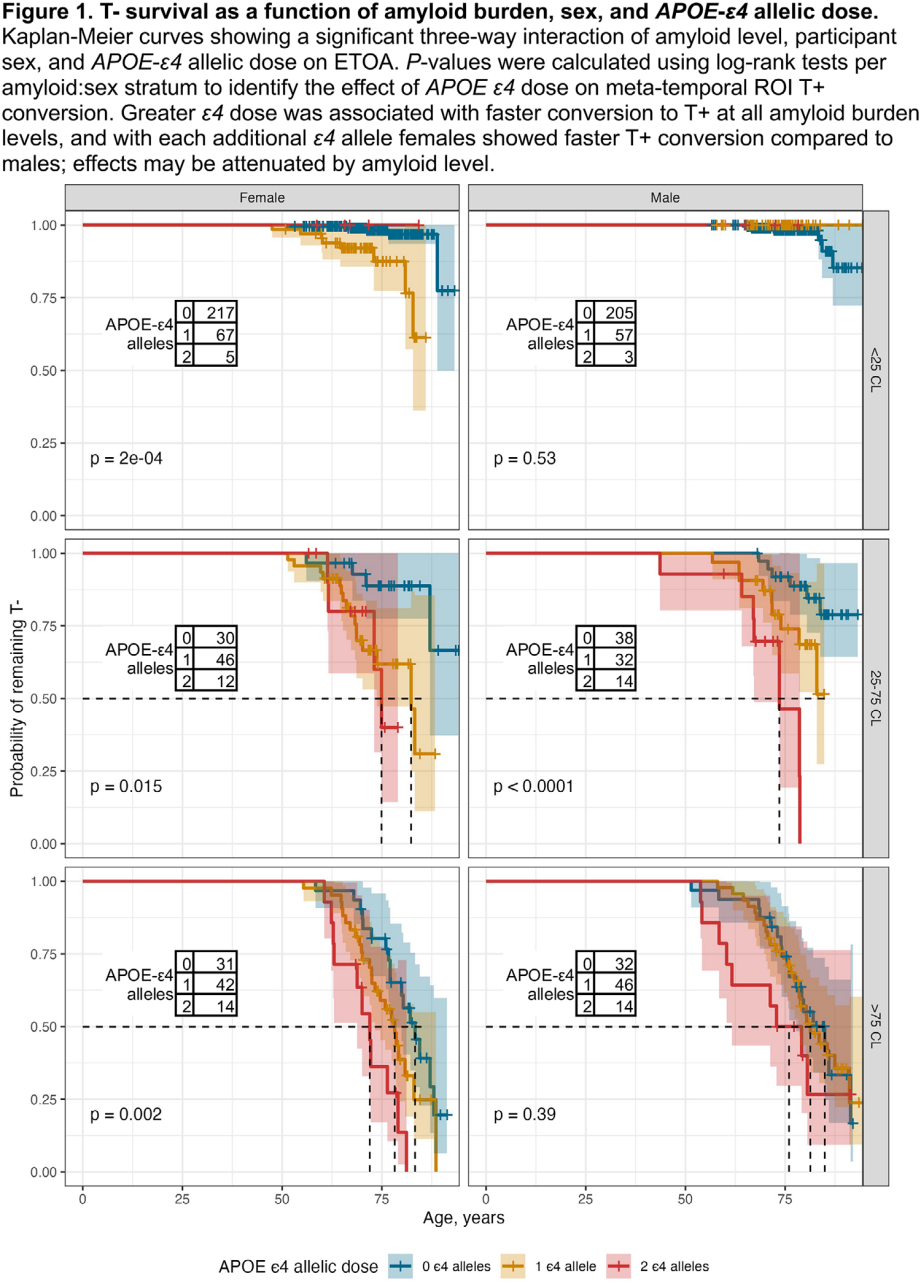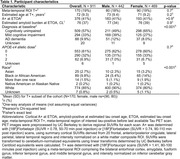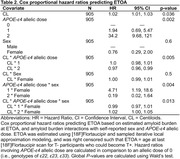# Amyloid burden and APOE modify sex differences in estimated meta‐temporal region tau onset age

**DOI:** 10.1002/alz.093647

**Published:** 2025-01-09

**Authors:** Margo B. Heston, Karly Cody, Jordan P Teague, Elena Ruiz De Chavez, Jacob Morse, Yuetiva Deming, Corinne D. Engelman, Richard J Chappell, Rebecca E. Langhough, Carey E. Gleason, Lindsay R. Clark, Megan L. Zuelsdorff, Tobey J. Betthauser

**Affiliations:** ^1^ Wisconsin Alzheimer’s Disease Research Center, University of Wisconsin School of Medicine and Public Health, Madison, WI USA; ^2^ Stanford University, Stanford, CA USA; ^3^ Wisconsin Alzheimer’s Institute, University of Wisconsin School of Medicine and Public Health, Madison, WI USA; ^4^ Wisconsin Alzheimer's Disease Research Center, University of Wisconsin School of Medicine and Public Health, Madison, WI USA

## Abstract

**Background:**

Recent advances in Alzheimer’s disease (AD) temporal biomarker modeling have revealed considerable heterogeneity in age at amyloid onset, and recently we and others identified sex and APOE differences in tau onset age and subsequent dementia development. Here we assessed whether amyloid burden, APOE‐e4 dose, and sex interact to predict estimated T+ onset age (ETOA).

**Methods:**

Alzheimer’s Disease Neuroimaging Initiative participants (N = 911, Table 1) underwent serial PET imaging to quantify global cortical amyloid ([18F]Florbetapir SUVR50‐70, [18F]Florbetaben SUVR90‐110) and meta‐temporal tau ([18F]Flortaucipir SUVR80‐100) burden. Cortical amyloid SUVRs were harmonized using linear transformation to Centiloids (CL). Sampled iterative local approximation (SILA) was used to model ETOA and, separately, to estimate amyloid CL burden at ETOA. Kaplan‐Meier survival curves and Cox regression were used to test CL*APOE‐e4*sex interaction effects on ETOA. Event time was ETOA for T+ individuals and age at last tau PET scan (right censored) for T‐ individuals who could become T+.

**Results:**

We observed a significant CL*APOE‐e4*sex interaction on ETOA (p = 0.013, Table 2). APOE‐e4 was associated with earlier ETOA in a dose‐dependent manner and effects were greater in females compared to males. ETOA differences between sexes and across APOE‐e4 were attenuated at higher amyloid burden, largely due to the increased presence of T+ across all APOE‐e4 statuses and both sexes (Figure 1). Main effects also revealed that higher cortical amyloid burden at ETOA was associated with earlier ETOA and greater T+ risk, as was greater APOE‐e4 dose (Table 2).

**Conclusions:**

These results suggest that amyloid burden, APOE‐e4, and sex may influence when temporal tau deposition becomes abnormal. Smaller sample size among APOE‐e4 carriers, and especially e4 homozygotes, limits the estimates in this cohort. Future extensions of this work will expand to a multicohort sample, to attempt replication of these findings and to investigate how these factors and their interaction predict time from amyloid and tau onset to dementia.